# Digitalization, Psychological Well-Being, and the Third-Level Digital Divide: Survey Study During the COVID-19 Pandemic in China

**DOI:** 10.2196/48195

**Published:** 2025-08-18

**Authors:** Fen Lin, Lei Jin, Xi Chen

**Affiliations:** 1 Department of Media and Communication City University of Hong Kong Hong Kong China (Hong Kong); 2 Department of Sociology The Chinese University of Hong Kong Hong Kong China (Hong Kong); 3 Department of Sociology and Social Policy Lingnan University Hong Kong China (Hong Kong)

**Keywords:** digital divide, health disparity, internet usage, digital inclusion, social inequality, China, COVID-19, psychological distress

## Abstract

**Background:**

While rapid digitalization has helped society cope with the uncertainty of the COVID-19 pandemic, will it bring health equity to the digitally disadvantaged? Limited studies have explored how individuals’ digital activities may impact their psychological well-being during the pandemic and whether these effects vary across different sociodemographic groups.

**Objective:**

This study aims to examine how individuals’ digital activities influence their psychological well-being and whether socioeconomic status moderates the relationship between digitalization and mental health.

**Methods:**

This study was based on a sample of 2170 residents surveyed in Hubei, the early epicenter of the pandemic in China, between March 23 and April 9, 2020. We first examined the main effects of online behavior and community e-group involvement on psychological distress. Then, we used ordinary least squares regression models to analyze the 2-way interaction effects between internet usage and socioeconomic status (SES) variables—education, occupation, monthly income, and urban/rural residence—on psychological distress.

**Results:**

First, the data reveal a pattern of digital divide during the pandemic. Participants with higher SES showed a greater propensity to use the internet for work and study purposes, as well as to engage in electronic groups (e-groups), compared with those with lower SES. By contrast, lower-SES respondents were more inclined to participate in entertainment and information-seeking activities than their higher-SES counterparts. Second, the data reveal the emergence of a third level of digital divide concerning psychological well-being. Specifically, the impact of online entertainment and communication activities on mental well-being varies based on employment status (b=–1.024, *P*=.03) and rural versus urban residence (b=–1.244, *P*=.046). These findings suggest that online entertainment and communication may have a more pronounced effect in reducing distress among individuals with lower SES than those with higher SES. Third, we observed a significant interaction between participation in community e-groups and rural versus urban areas (b=2.388, *P*=.047). This suggests that the impact of joining virtual communities on psychological distress is less pronounced among rural residents compared with their urban counterparts.

**Conclusions:**

The study illustrates how digital activities affect mental distress, providing evidence of the third-level digital divide in psychological well-being. First, the impact of digital activities on mental distress varied according to the type of internet usage. Second, online activities offered greater psychological benefits to individuals in lower social positions compared with those with higher social status. Third, digital inclusion in community e-groups helped alleviate psychological distress, but “digitalized social capital” provided more significant benefits to urban residents than to rural residents. These results highlight how the digital divide affects health inequalities and underscore the need for a more nuanced understanding of information and communications technology policies and their impacts in the post–COVID-19 world.

## Introduction

### Background

During the COVID-19 crisis, the world relied on social distancing measures to slow the spread of the pandemic. As a result, people increasingly turned to the internet and digital technology for daily activities, ranging from grocery shopping to formal international conferences, at an accelerated pace. However, it remains unclear whether and how such digitalization may exacerbate or mitigate existing health inequalities (see review by [1,2]). The widespread adoption of information and communication technology (ICT) has enhanced society’s ability to cope with the uncertainty brought on by this unforeseen crisis. Yet, comprehensive digitalization could further marginalize the digitally disadvantaged, deepening health and social inequalities [3]. Therefore, it is crucial to examine the health consequences of the digital divide.

Existing research on the digital divide has gradually shifted from focusing on the unequal distribution of access to ICT, known as “the first-level divide” [4], to examining ICT usage, skills, and literacy, referred to as “the second-level divide” [5]. More recently, research has begun exploring “the third-level divide”: the effects of digital technologies and digital capital on the life opportunities of different social strata [6]. In other words, the “third-level divide” refers to differences in people’s ability to use and access ICT to achieve offline results [7-9]. Although existing studies have examined who benefits from ICT in terms of offline outcomes, most discussions focus on the economic, social, political, institutional, and educational impacts of the digital divide (see review in [6,7,9]). Moreover, only a few studies have explored the connection between the digital divide and health outcomes [10-12]; however, the COVID-19 pandemic has brought health inequality back to the forefront.

This study examines the impact of digitalization on psychological well-being and the third-level digital divide, using data from a survey of 2170 residents in Hubei, the early epicenter of the pandemic in China, conducted between March 23 and April 9, 2020. To our knowledge, no other data are available that specifically examine the third-level digital divide in relation to psychological well-being during the peak of the pandemic in China.

More importantly, our findings and their applications contribute to at least three key areas, as the rapid digitalization of health, particularly driven by recent technological developments, extends beyond the pandemic. First, our study is among the first empirical efforts to provide clear evidence of the complex patterns of the third-level digital divide in health. The “third-level divide” has been an emerging theoretical concept in the digital divide literature. However, few studies have explored the link between the digital divide and health outcomes [10-12]. Our study not only calls for a more nuanced conceptual framework for understanding health equity but also provides a methodological foundation for comprehensively measuring the third-level divide. Second, our findings indicate that the impact of digitalization on health equity does not follow a simple, linear pattern where higher socioeconomic status (SES) always leads to better outcomes. The complex and intersecting patterns of the third-level divide may offer valuable insights for policy makers on how to enhance social inclusion and health equity in the era of rapid digitalization. Third, by incorporating the role of digital communities into our analysis, this study also enriches the existing research on psychological well-being and health disparities.

### Three Levels of Digital Divide

According to the Organisation for Economic Co-operation and Development (OECD), the digital divide refers to “differences between individuals, households, companies, or regions in terms of access to and use of ICT.” [13]. This divide reflects unequal technological opportunities. Conventional discussions of the first-level digital divide, often referred to as the access divide, typically focus on physical ICT connectivity and emphasize the binary distinction between the “haves” and the “have-nots” [4]. Recent studies have called for moving “beyond access” by focusing more on the second-level digital divide: the gap in skills and usage among ICT users. Researchers are working to understand the underlying factors contributing to differences between “knows vs know-nots” and “uses vs use-nots.” These factors range from technical tools, usage patterns, and support from social networks, to ICT experience [5,14-16], as well as cultural attitudes and values [17]. Recent research has shifted to the third level: the impact divide, which examines differences in the offline consequences of utilizing digital resources. In other words, even with equal digital access and similar skills, users may not achieve the same offline returns from their internet use [6,9,18,19]. Furthermore, individuals who derive high offline returns from internet use may experience retroactive benefits, as increased socioeconomic resources can further enhance their digital access and skills [9].

China has undergone a significant digital transformation in the past 2 decades. By the end of 2020, there were 989 million internet users in the country [20], with 932 million accessing the internet via mobile phones. Alongside the accelerated development of the internet, notable disparities in access exist between rural and urban regions, as well as among different genders, education levels, and income groups [21-23]. In addition to access divides, the second-level digital divide in internet usage across different age groups, genders, and socioeconomic positions is greater than the access divide among these groups in China [24]. However, existing studies on the digital divide rarely address the third-level divide, and the offline consequences of internet use remain underexplored [25]. Thus, in response to the growing call to focus on the third-level divide (eg, [8]), our study investigates this divide, particularly the link between the digital divide and health inequality.

### ICT Use and Psychological Well-Being

Existing studies have outlined various mechanisms through which ICT use can impact health, but empirical investigations have yielded mixed findings. The initial media and public reactions to the internet were largely negative, often characterized as apocalyptic [26]. Scholars have articulated the *displacement mechanism,* suggesting that internet usage can be detrimental to users’ social and psychological well-being because it encourages reliance on online relationships at the expense of offline ones [27-29]. This conclusion has fueled anti-internet sentiment in popular media [30,31].

Other scholars have proposed the *simulation mechanism,* which posits that communication technologies can reduce feelings of loneliness by enhancing existing relationships and facilitating the formation of new ones [32-36]. Additionally, ICT usage has improved public health emergency responses and enhanced the effectiveness of disease surveillance [37,38]. Thus, ICT enables people to access health-related information, make informed decisions, and adopt healthy behaviors [5]. This mixed evidence highlights the need to consider both the displacement and stimulation functions of ICT usage simultaneously, as these 2 mechanisms may not be mutually exclusive [29].

During the pandemic, internet use may have both beneficial and harmful consequences for mental health. On the one hand, it provides users with up-to-date information about the outbreak and guidelines for protective measures, which is crucial for combating the pandemic. Additionally, the internet enables individuals to work and study remotely and helps maintain social connections with family and friends they cannot meet in person, thereby mitigating the psychological impacts of isolation. On the other hand, excessive exposure to pandemic-related information, particularly misinformation and disinformation, can lead to an “infodemic,” which may increase psychological distress [39-41].

Such inconsistent findings suggest that the impact of internet usage on mental well-being may vary depending on specific online activities. However, most research on the psychological consequences of internet use tends to emphasize the overall number of hours spent online (eg, [10,42]). Gaps remain in examining how the effectiveness of internet use differs based on its various purposes. For example, information seeking appears to be the most universally developed skill, while commercial transactions represent the widest divide, possibly due to the relatively higher barriers involved in online transactions [24]. Additionally, past research has often focused on the mental health effects of internet addiction, leaving the health impacts beyond addiction understudied.

Thus, we hypothesize the following:

- H10: Hubei residents with higher internet usage tended to experience less psychological distress during Hubei’s lockdown period.
- H1a: Hubei residents with higher internet usage tended to experience more psychological distress during Hubei’s lockdown period.
- H2: The associations between internet usage and psychological distress vary by the types of internet activities.

### Prior Studies on the Digital Divide and Psychological Well-Being

Different strands of research have produced conflicting predictions regarding whether ICT usage may exacerbate or alleviate existing health inequalities. The theory of fundamental causes of health examines social determinants and posits that as health-promoting innovations become available, individuals with greater social privilege are more likely to adopt and benefit from these innovations, potentially widening socioeconomic gaps in health [43,44]. This theory suggests that individuals in better socioeconomic conditions have greater access to resources that enable them to effectively use ICT and derive more health-promoting benefits [25,45,46]. Several empirical studies on the consequences of ICT usage have shown that privileged users (eg, highly educated individuals) use the internet more effectively and gain more benefits than those with lower social status [9,22,47]. These findings indicate a widening gap based on existing inequalities [5,48].

However, studies on ICT applications in health promotion suggest that ICT usage reduces the costs of accessing information and health services, benefiting socially disadvantaged individuals who previously lacked the resources to access these resources [1,2]. This line of argument suggests that ICT usage may help reduce socioeconomic disparities in health and well-being. Empirical evidence from Western societies has confirmed that digital technology usage can improve mental health for socioeconomically disadvantaged groups [2,42,49]. Studies in China have also found that ICT adoption may alleviate socioeconomic disparities in mental health [50-52].

During the pandemic, as social distancing measures heightened digital lifestyles, the impacts of the digital divide became more pronounced in the varying quality of ICT usage among different societal strata [53]. On the one hand, low-SES individuals have access to fewer offline material resources and social capital; thus, the resources and connections they obtain online may play a particularly crucial role in enhancing their well-being. On the other hand, utilizing the benefits of digitalization requires both material and cognitive resources. Consequently, it is the wealthier, better-educated individuals with higher social status who are more able to take advantage of expanding digital opportunities and mitigate potential harms, leaving poorer and less educated individuals at a disadvantage. A recent study using 2-wave panel data from Hubei residents found that better-educated respondents were more likely to obtain information from social media [54]. Therefore, we aim to explore health disparities along the digital divide, as it has created new dimensions of social segmentation that are intertwined with traditional economic, social, and cultural inequalities. We hypothesize the following:

- H3_0_: The positive association between internet usage and psychological well-being is more pronounced among those with higher SES, thereby widening existing health disparities.
- H3_a_: The positive association between internet usage and psychological well-being is more pronounced among those with lower SES, thereby reducing existing health disparities.
- H4: The associations between e-community engagement and psychological well-being vary by SES.

Two additional caveats are worth noting. Most research on the digital divide and its impact on health disparities has not sufficiently considered neighborhood and community characteristics [11]. Sociologists have demonstrated the value of community in enhancing individual well-being [55-57]. In the context of China’s antipandemic measures, the significance of digital communities organized by neighborhood committees has been well documented. A qualitative study of Wuhan residents during the COVID-19 outbreak found that WeChat, the most widely used social media app in China, served as essential infrastructure for meeting their practical, emotional, and medical needs during the 76-day lockdown [58]. WeChat groups (*qun*) facilitated information dissemination and addressed residents’ various requests, ranging from group purchases (*tuangou*) to medical appointments, helping people cope with uncertainty and fear. Communication in e-groups helps maintain social connectedness despite physical isolation, creating a new form of social capital known as “virus-combat social capital” [59]. Therefore, this study also incorporates engagement in community e-groups via WeChat (Tencent Holdings Limited) and QQ groups (Tencent QQ; Shenzhen Tencent Computer System Co., Ltd/Tencent Holdings Ltd) into the analysis.

Additionally, studies on the third-level divide often rely on self-perceived measures of beneficial outcomes (eg, “online entertainment made me feel happier”). Although these measures assess perceived psychological well-being (eg, “feeling happier”), they also subjectively evaluate the causal relationship between online activities and mental status, which needs to be tested with empirical data. To avoid such compounding measurements and to estimate the health impacts of the digital divide, our study adopts a more objective measure of psychological health: the 12-item Chinese Health Questionnaire scale (CHQ-12). The CHQ-12 is widely used to assess nonpsychotic mental health issues in the general population.

## Methods

### Data

The first case of COVID-19 was recorded in December 2019 in Wuhan, the capital of Hubei province, China. On January 23, 2020, a strict lockdown was implemented in Wuhan and 16 nearby cities in Hubei, which was eased on March 23 and ultimately lifted on April 8, 2020. During the lockdown, it is likely that Hubei residents experienced fear, panic, and psychological distress and relied heavily on online resources. The data were collected during the mitigation period of the pandemic in Hubei, when lockdowns were gradually eased and ultimately lifted. This created a unique opportunity to examine the internet usage and psychological well-being of Hubei residents during the lockdown.

The data for this study were obtained from the “Public Attitude Toward the COVID-19 Pandemic in Hubei Province” survey conducted by the China Academy of Science and Technology Development Strategy, the Social Policy Research Institute at Renmin University, and the Institute of Sociology of the Chinese Academy of Social Sciences. The survey was conducted from March 23 to April 9, 2020.

The survey targeted all eligible residents aged 18-80 years living in both urban and rural areas of Hubei. It was conducted online via a professional survey platform in China, supplemented by phone surveys conducted by trained research assistants. We acknowledge the limitation that our sample may not fully represent the general population of Hubei, as the survey was primarily administered through online platforms. However, during crises such as the COVID-19 pandemic, online surveys often represent the most efficient and feasible means of data collection. To address this limitation, we supplemented our online survey with a telephone survey targeting individuals with limited internet access, typically older adults residing in rural areas with lower SES.

To ensure the reliability of the online survey, we implemented several quality control measures. Before launching the survey, we conducted a pilot test with a small group of participants to identify and address potential issues related to the clarity, comprehension, and functionality of the survey instrument. In terms of survey design, the questions were meticulously crafted by a team of sociologists, psychologists, and public health experts. They were designed to be clear, concise, and unambiguous to minimize confusion or misinterpretation. Additionally, skip logic and validation checks were incorporated to ensure accurate and consistent responses. To enhance the reliability of the online survey, attention check questions were strategically inserted at various points throughout the questionnaire. An example of an attention check question was “Please select ‘Strongly Agree’ for this question.” Participants who did not respond accurately to these questions were flagged for potential exclusion from the dataset. The final sample size consisted of 2170 respondents after eliminating cases with at least one missing value.

### Measurements

#### Psychological Distress

This was assessed using the CHQ-12 scale [60]. The CHQ-12 is a modified version of the General Health Questionnaire [61], incorporating culturally relevant items for Chinese societies. Among the 3 available versions of the CHQ (CHQ-60, CHQ-30, and CHQ-12), the CHQ-12 has been deemed the most effective [62]. It has been validated in the general population of mainland China [63,64], as well as among Taiwanese individuals [60,65] and Chinese populations abroad [66,67]. The scale consists of 10 negatively worded items (eg, “Have you been experiencing headaches or pressure in your head?”) and 2 positively worded items (eg, “Have you been getting along well with your family or friends recently?”), with responses ranging from 0 (not at all) to 3 (very often). The responses for the positive items were reverse-coded so that higher scores indicated greater levels of mental distress. The total score was calculated by summing the scores of all 12 items, and the scale demonstrated good internal validity (Cronbach α=0.8).

#### Socioeconomic Status

We included 4 SES variables in the analysis: *education* (≤middle school vs ≥high school; education was categorized into 2 groups: those who finished high school or more and those with less education. Considering China’s 9-year compulsory schooling, people with less than a middle school education may encounter distinct socioeconomic challenges, potentially exacerbating the digital divide and its impact on mental health among Hubei residents during the COVID-19 pandemic. Similar measures of education have been used in previous studies on education and well-being in China [68]), *employment status* (full/part-time employment vs other), *monthly personal income* (≤2000 yuan vs >2000 yuan; 1 yuan=US $0.14), and *location* (rural vs urban).

#### Internet Usage

Following previous studies [9,69-71] that classify digital activities based on specific types or purposes of use, we categorized internet usage into 4 conceptually distinct types: (1) information seeking (eg, reading news); (2) study/work; (3) entertainment/communication; and (4) economic activities (eg, purchasing products, investment, and financial management). Participants rated their usage frequency on a scale from 0 (none) to 4 (daily).

#### Engagement in Community E-Groups

This was measured by whether respondents participated in e-groups, such as WeChat/QQ groups organized by the official neighborhood committee or residents during the COVID-19 pandemic (yes/no).

In addition to the SES indicators mentioned earlier, the analysis controlled for several variables, including age (≤25, 26-35, 36-50, or ≥51 years), sex (male vs female), residential location (Wuhan vs non-Wuhan), and housing type (luxury apartments, regular apartments, public/low-cost housing, or rural/urban village). Additionally, the study controlled for participants’ risk exposure (ie, whether they, their family members, neighbors, or residents in the same community had a confirmed COVID-19 infection) and risk perception (ie, their subjective probability of recently getting infected with COVID-19).

### Statistical Analysis

First, we presented descriptive statistics of digital activities (information seeking, work/study, entertainment/social, commerce, and e-community involvement) in Table 1, along with group differences in online behavior based on socioeconomic factors (education, employment, monthly income, and rural/urban residence). Group differences were assessed using 2-tailed *t* tests or *χ*^2^ tests. We then applied ordinary least squares regression models to examine the relationship between internet use and psychological distress. We analyzed the main effects of online behavior and community e-group involvement on mental distress, controlling for demographic and socioeconomic variables. Additionally, we investigated whether 4 socioeconomic variables (education, occupation, monthly income, and urban/rural residence) moderated the association between internet usage and psychological distress. To achieve this, we assessed the interaction effects between internet usage and each SES factor on psychological distress. All analyses were conducted using Stata 16.0 (StataCorp), with a *P* value of .05 set as the threshold for statistical significance.

### Ethical Considerations

Ethics approval for the study was obtained from the Ethics Committee of the Chinese Academy of Social Sciences (approval number CHN-2153, 18/0020), in accordance with the principles outlined in the Declaration of Helsinki. Informed consent was obtained from all survey participants, with written consent collected from those who completed the online survey. Oral consent was secured from participants who responded via telephone, with the interviewer signing a document confirming adherence to proper procedures for obtaining verbal informed consent. No incentives were provided to participants. Study data were anonymized to ensure the privacy and confidentiality of all participants.

## Results

### Respondents’ Characteristics

Most respondents were female (1116/2170, 51.43%) and aged below 35 years (1349/2170, 62.17%). A large majority had a high school education or above (1907/2170, 87.88%) and were employed (1800/2170, 82.95%). Over half reported a monthly income exceeding 2000 yuan (1321/2170, 60.88%) and lived in urban areas (2003/2170, 92.30%), with nearly 75% (1622/2170, 74.75%) residing outside of Wuhan. Regarding housing, of the 2170 respondents, 910 (41.94%) lived in regular apartments, about 84 (3.87%) in luxury housing, 854 (39.35%) in public or low-rent housing, and the remaining 322 (14.84%) in rural or urban villages.

The mean score of psychological distress was 10.71 (out of a theoretical range of 0-36), which is only slightly lower than that reported by survivors of the 2008 Wenchuan earthquake, a devastating event that struck the central region of Sichuan province in China [72].

### Who Is Digitally Disadvantaged?

Table 1 presents the pattern of internet usage stratified by SES. Among the different types of online behavior, respondents most frequently engaged in information seeking (mean 3.54, SD 0.80), followed by entertainment and communication activities (mean 3.34, SD 0.78), work or study (mean 2.97, SD 0.99), and economic activities (mean 2.27, SD 1.01). Additionally, approximately 1781 out of the 2170 (82.07%) respondents reported having joined e-groups organized by neighborhood committees or residents, while the remaining 389 (17.93%) indicated that they had not participated in any community e-groups.

**Table 1:** Internet usage and e-community involvement by socioeconomic status (n=2170).^a^

Variables		Total	Education				Employment				Monthly income				Location		
			≤Middle school (n=263)	≥High school (n=1907)	*t* test/chi-square (*df*)	Unemployed/peasant/student/retired (n=370)		Employed (n=1800)	*t* test/chi-square (*df*)	≤2000 yuan^b^ (n=849)		>2000 yuan (n=1321)	*t* test/chi-square (*df*)	Rural (n=167)		Urban (n=2003)	*t* test/chi-square (*df*)
**Online behavior**																	
	Information-seeking	3.54 (0.80)	3.55 (0.77)	3.54 (0.81)	0.15 (2168)	3.51 (0.85)		3.55 (0.79)	–0.95 (2168)	3.54 (0.86)		3.55 (0.76)	–0.44 (2168)	3.74 (0.58)		3.53 (0.82)	3.23^c^ (2168)
	Work/ study	2.97 (0.99)	2.95 (1.00)	2.98 (0.99)	–0.35 (2168)	2.89 (1.07)		2.99 (0.97)	–1.73 (2168)	2.90 (1.05)		3.02 (0.94)	–2.81^c^ (2168)	2.87 (1.06)		2.98 (0.98)	–1.40 (2168)
	Entertainment/social	3.34 (0.78)	3.24 (0.78)	3.35 (0.78)	–2.19^d^ (2168)	3.40 (0.75)		3.33 (0.79)	1.85^e^ (2168)	3.35 (0.78)		3.34 (0.78)	0.32 (2168)	3.31 (0.82)		3.34 (0.78)	–0.50 (2168)
	Commerce	2.27 (1.01)	2.24 (1.06)	2.28 (1.00)	–0.54 (2168)	2.19 (1.12)		2.29 (0.99)	–1.73 (2168)	2.15 (1.08)		2.36 (0.96)	–4.65^f^ (2168)	1.93 (1.11)		2.30 (1.00)	–4.63^f^ (2168)
**E-community involvement**																	
	Joined community e-groups	82.07	82.07	82.13	*0.001*^g^ (1)	78.65		82.78	*3.56* (1)	77.27		85.16	*21.90*^f^ (1)	79.64		82.28	*0.73* (1)
	Did not join community e-groups	17.93	17.93	17.87	N/A	21.35		17.22	N/A^h^	22.73		14.84	N/A	20.36		17.72	N/A

^a^Values are presented as either mean (SD) or %.

^b^1 yuan=US $0.14.

^c^*P*<.01.

^d^*P*<.05.

^e^*P*<.1.

^f^*P*<.001.

^g^Chi-square values are italicized.

^h^N/A: not applicable.

The extent to which social inequality translates into digital inequality varies by type of internet usage. Respondents with a higher monthly income (>2000 yuan) were more likely to use the internet for study and work compared with their low-income counterparts. Additionally, respondents with higher SES, such as urban residents and those with higher monthly incomes, were more likely to engage in online economic activities. Similarly, better-educated respondents (ie, those with a high school education or above) were more likely to engage in online entertainment and communication activities compared with those with a middle school education or below. Interestingly, rural respondents were found to read news online more frequently than their urban counterparts, which was an unexpected finding.

Regarding digital inclusion, as measured by membership in various community e-groups, the digital divide was correlated with income; the proportion of respondents joining any e-groups was substantially higher among high-income individuals.

### Impacts of the Digital Divide on Psychological Well-Being

Table 2 presents the ordinary least squares regression results examining the associations between sociodemographic variables, internet usage, and psychological distress. Model 1 explores the relationship between online behavior and psychological distress. The results indicate that certain online activities—specifically information seeking, studying/working, and entertainment/communication—were negatively associated with psychological distress. Conversely, engagement in online economic activities was positively correlated with mental distress.

**Table 2:** Associations between online usage and e-group involvement and psychological distress (N=2170).^a^

Associations		Model 1, coefficient (SE)	Model 2, coefficient (SE)
**Online behavior**			
	Information seeking	–1.076^b^ (0.171)	N/A^c^
	Study/work	–0.461^d^ (0.144)	N/A
	Entertainment/socializing	–0.422^e^ (0.177)	N/A
	Commerce	0.684^b^ (0.140)	N/A
**E-group involvement (reference: did not join any e-groups)**			
	Joined community e-groups	N/A	–1.146^b^ (0.341)

^a^All models were controlled for sociodemographic covariates, including age, sex, education, employment status, monthly income, area, and housing types.

^b^*P*<.001.

^c^N/A: not applicable.

^d^*P*<.01.

^e^*P*<.05.

Model 2 investigates the impact of community e-groups on residents’ mental health during the pandemic. The findings indicate that respondents who participated in neighborhood e-groups experienced lower levels of mental distress compared with those who did not join any groups. Therefore, both H1_0_ and H2 are supported.

Table 3 explores how the impacts of internet usage on psychological distress differ across various socioeconomic groups. Model 1 analyzes the interaction between education and internet use concerning distress levels. A marginally significant negative interaction was found between online entertainment/communication activities and education (*b*=–1.038, *P*=.06), indicating that these online activities are more effective in reducing distress for individuals with lower education compared with those with higher education. Similarly, unemployed respondents reported lower levels of psychological distress when using the internet for entertainment and communication compared with their employed counterparts (model 2: *b*=–1.024, *P*=.03). Additionally, model 3 revealed a negative interaction between online entertainment/communication and rural/urban residence (*b*=–1.244, *P*=.046), indicating that rural respondents may experience greater reductions in psychological distress from participating in online entertainment/communication. These findings consistently demonstrate that individuals with lower SES benefit more from engaging in online entertainment and communication than those with higher status, thereby supporting H3_a_.

Additionally, we found a significant interaction between participation in community e-groups and rural/urban residence, as indicated in model 3 (*b*=2.388, *P*=.047). This suggests that the impact of joining virtual communities on psychological distress is less pronounced among rural residents compared with urban residents. The interactions between participation in virtual communities and other SES indicators—such as education (*P*=.49), and employment status (*P*=.80)—were not significant. Thus, H4 is supported.

**Table 3:** The interactive effects of online usage and socioeconomic status on psychological distress (N=2170).^a^

Interactive effects		Model 1, coefficient (SE)	Model 2, coefficient (SE)	Model 3, coefficient (SE)
**Online behavior**				
	Information seeking	–0.972^b^ (0.182)	–1.068^b^ (0.190)	–1.060^b^ (0.175)
	Study/work	–0.491^c^ (0.152)	–0.364^d^ (0.160)	–0.464^c^ (0.150)
	Entertainment/communication	–0.301 (0.187)	–0.278 (0.193)	–0.313^e^ (0.184)
	Commerce	0.788^b^ (0.149)	0.765^b^ (0.156)	0.678^b^ (0.146)
**E-group involvement** **(reference: did not join e-groups)**				
	Joined e-groups	–0.933^c^ (0.359)	–1.083^c^ (0.374)	–1.214^b^ (0.352)
**Education (reference:** **≥** **high school)**				
	≤Middle school	6.245^c^ (2.405)	–0.261 (0.398)	–0.309 (0.398)
**Employment status (reference: employed)**				
	Unemployed/peasant/retired/student	0.298 (0.363)	4.728^d^ (2.051)	0.310 (0.363)
**Monthly income (reference: >2000** **yuan^f^)**				
	≤2000 yuan	0.377 (0.273)	0.371 (0.274)	0.383 (0.273)
**Area (** **reference:** **urban)**				
	Rural	0.708 (0.482)	0.750 (0.482)	–0.188 (3.649)
**Online behavior** **×** **education**				
	Information seeking × ≤middle school	–0.637 (0.527)	N/A^g^	N/A
	Study/work × ≤middle school	0.413 (0.451)	N/A	N/A
	Entertainment/socializing × ≤middle school	–1.038 (0.543)	N/A	N/A
	Commerce × ≤middle school	–0.683 (0.419)	N/A	N/A
**E-group involvement** **×** **education**				
	Joined e-groups × ≤middle school	–0.712 (1.024)	N/A	N/A
**Online behavior** **×** **employment status**				
	Information seeking × unemployed/peasant/retired/student	N/A	0.149 (0.425)	N/A
	Study/work × unemployed/peasant/retired/student	N/A	–0.311 (0.365)	N/A
	Entertainment/socializing × unemployed/peasant/retired/student	N/A	–1.024^d^ (0.484)	N/A
	Commerce × unemployed/peasant/retired/student	N/A	–0.338 (0.342)	N/A
**E-group involvement** **×** **employment status**				
	Joined e-groups × unemployed/peasant/retired/student	N/A	0.211 (0.849)	N/A
**Online behavior** **×** **urban/rural area**				
	Information seeking × rural	N/A	N/A	0.384 (0.838)
	Study/work × rural	N/A	N/A	0.376 (0.514)
	Entertainment/socializing × rural	N/A	N/A	–1.244^d^ (0.625)
	Commerce × rural	N/A	N/A	0.301 (0.483)
**E-group involvement** **×** **rural/urban area**				
	Joined × rural	N/A	N/A	2.388^d^ (1.203)
	Constant	12.346^b^ (0.970)	12.400^b^ (0.992)	13.064^b^ (0.948)

^a^All models controlled for additional sociodemographic variables, including age, sex, and housing types.

^b^*P*<.001.

^c^*P*<.01.

^d^*P*<.05.

^e^*P*<.1.

^f^1 yuan=US $0.14.

^g^N/A: not applicable.

## Discussion

### Principal Findings and Comparison With Prior Work

Our study offers detailed evidence on whether the rapid digitalization during the COVID-19 pandemic contributes to social inclusion and health equity. Specifically, we found that different forms of internet usage had varying effects on the psychological well-being of Hubei residents during the pandemic. Participants who used the internet for information seeking reported lower levels of mental distress, which contrasts with previous studies on infodemic effects [40,73]. It is possible that our study’s measurement of general information-seeking behavior, rather than COVID-19–specific information seeking, contributed to this inconsistency. Future research should further explore the impact of both general and COVID-19–specific information-seeking behaviors on citizens’ psychological well-being during crises.

Additionally, engaging in online study or work, as well as communication or entertainment, could reduce mental distress during the pandemic. Given the restrictions on offline interactions during the lockdown, communicating with friends and family via the internet can enhance perceived social support, decrease feelings of loneliness and social isolation, and subsequently improve mental health. Similarly, online entertainment can lead to positive well-being outcomes, serving as a coping mechanism to alleviate feelings of depression and isolation while helping individuals to “recharge their batteries” [74]. Additionally, remote work and learning during the pandemic allow people to maintain connections with colleagues and peers, even when physically separated. Using the internet for professional and academic pursuits can help establish a regular daily routine, which is essential for managing stress and improving mental health during a crisis [75]. Conversely, engaging in online economic and financial activities, such as shopping and investment, has been associated with increased mental distress. The economic insecurity and unpredictability during the pandemic may have contributed to the heightened distress experienced by individuals involved in these online economic activities. Additionally, prior research suggests that using online shopping as a coping strategy during stressful times can be detrimental to mental health and is associated with higher levels of depression [76,77]. While online shopping became a primary means for people to acquire groceries during the lockdown, scholars recommend establishing financial and time limits to prevent compulsive and addictive behaviors [78].

Second, the impact of internet use on citizens’ psychological well-being was more pronounced in reducing mental distress among respondents with lower SES (ie, those with ≤middle school education, rural residents, and the unemployed). Although users with high SES are more likely to utilize the internet productively, they may not rely on it as much to overcome social, economic, and informational challenges. By contrast, the internet can assist low-SES users in transcending financial limitations, accessing low-cost information, and engaging in various social activities, all of which are critical for mental well-being [79]. Therefore, the health benefits of internet use are more pronounced among those in lower socioeconomic positions compared with their high-SES counterparts.

Third, our findings indicated that individuals who joined community e-groups during the pandemic experienced better mental health compared with those who did not. During the lockdown, neighborhood committees played a crucial role in supporting residents and enforcing lockdown measures. These e-groups enhanced the mobilization capacity of local organizers and provided effective channels for information distribution and feedback, which can improve residents’ mental health and help them cope with stressful situations [58,80]. Moreover, the psychological benefits of participating in these e-groups seemed to be stronger among urban residents than their rural counterparts. This may be attributed to the faster spread of the virus and higher infection rates in urban China [81]. Additionally, stricter enforcement of quarantine measures in urban areas led to greater reliance on virtual communities to meet daily needs. It is also possible that offline urban neighborhoods are less cohesive than rural village communities, making online communities play a more complementary role for urban residents.

Our results also have implications for ICT policies. Efforts to provide access to hardware have led to significant gains, facilitating a transition from inequality to equality in digital terms. However, as rapid digitalization becomes the new normal after COVID-19, traditional digital divides may evolve into a triple divide: the “access divide,” the “ICT literacy divide,” and the “impact divide” [82,83]. To address the multiple layers of the digital divide, ICT policy makers face the challenging task of ensuring both equality and equity. Recently, the General Office of the State Council in China issued a plan aimed at effectively addressing the difficulties faced by older adults in using smart technology. This plan sets goals to significantly enhance the level and accessibility of smart services for older adults by the end of 2022 and to establish long-term measures to mitigate digital inequality [84]. In other words, the current digital landscape has adopted a more complex structure, where digital inequality is intricately woven into the fabric of social inclusion. Accordingly, ICT policies must reflect a more nuanced understanding of the third-level implications of the digital divide in the post–COVID-19 new normal.

### Limitations

Several limitations need to be addressed. First, as a cross-sectional study, this research cannot establish causal relationships among the investigated variables. Future longitudinal studies are essential for exploring the causal link between online behavior and psychological well-being during the pandemic. Second, the nonrepresentative sample in this study was primarily obtained through online surveys, potentially excluding individuals without internet access. Consequently, our findings pertain mainly to the second and third levels of the digital divide. However, given the impracticality of conducting in-person surveys during lockdowns, online surveys provide a practical and efficient method for data collection. Moreover, to reach individuals with limited internet access, we supplemented our online survey with a telephone survey. Future research could adopt diverse sampling approaches to obtain a population-based representative sample. Additionally, current methods for measuring the digital divide are relatively simplistic and may not adequately capture the complex and dynamic nature of the digital divide in the current ICT environment. Our research faces similar measurement challenges, highlighting the need for further development of more comprehensive measures of the digital divide.

### Conclusions

Based on survey data collected in Hubei, China, we assessed how digital activities and digital inclusion influence psychological well-being, and how the socioeconomic conditions of Hubei residents moderate this influence during the COVID-19 pandemic. Our findings indicate that most online activities and digital social inclusion positively contributed to individual psychological well-being during the lockdown. However, digital activities provided greater psychological benefits to individuals in lower social positions, while digitalized social inclusion offered more psychological support to urban residents compared with their rural counterparts. These findings not only enhance current theories on the digital divide but also offer valuable insights for policy makers on promoting social and health equity in the emerging “new normal” of a post–COVID-19 society.

## Abbreviations

CHQ: Chinese Health Questionnaire
ICT: information and communication technology
OECD: Organisation for Economic Co-operation and Development
SES: socioeconomic status

## Data Availability

The datasets analyzed during this study are not publicly available yet due to data protection regulations. However, the datasets are available from the corresponding author (FL) upon reasonable request.
